# Wie erleben und bewältigen Lehrende der berufsbezogenen Weiterbildung Folgen der Corona-Pandemie?

**DOI:** 10.1007/s40955-021-00193-4

**Published:** 2021-11-19

**Authors:** Susanne Wißhak, Sabine Hochholdinger

**Affiliations:** Universität Konstanz, Konstanz, Deutschland

**Keywords:** Lehrende in der Weiterbildung, Trainerinnen und Trainer, Corona-Pandemie, Individuelle Professionalisierung, Kompetenzentwicklung, Qualitative Inhaltsanalyse, Teachers in continuing education, Trainers, Corona pandemic, Individual professionalization, Development of competences, Qualitative content analysis

## Abstract

Die qualitative Studie geht der Frage nach, wie Lehrende in der berufsbezogenen Weiterbildung die Folgen der Corona-Pandemie im Hinblick auf die damit einhergehenden Unsicherheiten und professionellen Anforderungen erlebten und bewältigten. Ausgehend von Kompetenzmodellen für Lehrende in der Weiterbildung wurden die Veränderungen ihrer beruflichen Situation betrachtet, die veränderten Anforderungen mit Blick auf die Digitalisierung sowie ihre Selbstregulations- und Bewältigungsstrategien. Zu diesem Zweck werteten wir 22 textbasierte Beiträge einer Webparade vom Spätsommer 2020 mittels der qualitativen Inhaltsanalyse aus. Es zeigte sich, dass die Lehrenden nach anfänglicher Verunsicherung proaktiv und pragmatisch reagierten und die Digitalisierung als Lerngelegenheit annahmen. Für den Erwerb der erforderlichen Kompetenzen scheinen sie sich mittels Online-Trainerausbildungen und auf informellen Wegen fortzubilden. Aus didaktischer Sicht scheint die Digitalisierung Vorteile für bedarfsorientierte und wirksame Weiterbildung zu bieten, bei gleichzeitigen Schwierigkeiten, die aus der physischen Distanz vor allem für die Interaktion und Beziehungsgestaltung resultieren. Implikationen für die individuelle Professionalisierung der Lehrenden unter Pandemiebedingungen werden diskutiert.

## Abstract (en)

This qualitative study explores the question of how trainers in work-related continuing education experienced and coped with the consequences of the COVID-19-pandemic in terms of the uncertainties and professional demands that accompanied it. Based on competency models for teachers in continuing education, we looked at the changes in their professional situation, the changed requirements with regard to digitalization, and their self-regulation and coping strategies. For this purpose, we evaluated 22 text-based contributions of a web parade from late summer 2020 using qualitative content analysis. We found that after initial uncertainty, trainers responded proactively and pragmatically, embracing digitization as a learning opportunity. To acquire the necessary competencies, they appear to use online trainer training and informal channels. From a didactic point of view, digitization seems to offer advantages for demand-oriented and effective continuing education, with simultaneous difficulties resulting from physical distance, especially for interaction and relationship building. Implications for the individual professionalization of trainers are discussed.

## Einleitung

Ab Mitte März bis Anfang Mai 2020 durften in Deutschland aufgrund der Corona-Pandemie keine Weiterbildungsveranstaltungen in Präsenz angeboten werden. Auch später waren Präsenzveranstaltungen zeitweise nicht oder nur eingeschränkt möglich. Es ist anzunehmen, dass dies einen kritischen und bedeutsamen Umbruch für die Lehrenden in der betrieblichen und beruflichen Weiterbildung bedeutete, sowohl für ihre Arbeitsbedingungen als auch für ihre Lernangebote.

Die vorliegende Untersuchung befasst sich mit der Wahrnehmung und Bewertung der Corona-Pandemie durch die Lehrenden in der betrieblichen und beruflichen Weiterbildung, also durch sogenannte Trainerinnen und Trainer, die überwiegend als Selbständige arbeiten und auf Aufträge von Unternehmen angewiesen sind (Wißhak et al. 2020). Diese Personen werden in größeren Befragungen zur Corona-Pandemie, die sich zumeist mit Weiterbildungsorganisationen beschäftigen, selten mitberücksichtigt. Dabei interessierte uns vor allem die individuelle Professionalisierung i.S. des individuellen Umgangs mit neuen Kompetenzanforderungen der Lehrenden (Nittel und Seltrecht 2008). Neben den unmittelbaren beruflichen Auswirkungen der Pandemie wurden die didaktischen Anforderungen und benötigten Kompetenzen, die sich aus der Digitalisierung der Weiterbildungsangebote ergeben, untersucht, sowie die Selbstregulation und Bewältigung im Umgang mit den pandemiebedingten beruflichen Herausforderungen.

## Forschungsstand und Entwicklung der Forschungsfragen

Der vorliegende Artikel behandelt subjektiv wahrgenommene Anforderungen an die individuelle professionelle Kompetenzentwicklung (Nittel und Seltrecht 2008), speziell der Lehrenden in der betrieblichen und beruflichen Weiterbildung, weniger die kollektive und institutionalisierte Professionalisierung (Schrader und Loreit 2018). Empirische Befunde bezüglich des Personals in der Weiterbildung enthält u. a. der gleichnamige wb-personalmonitor (Autorengruppe wb-personalmonitor 2016), einen Überblick über Zertifizierungen für Lehrende im deutschsprachigen Raum gibt Gruber (2018).

Moraal (2018) stellt heraus, dass das berufsbezogene Weiterbildungspersonal trotz des hohen Anteils betrieblicher und beruflicher Weiterbildung an der gesamten Weiterbildung im Professionalisierungsdiskurs lange Zeit kaum berücksichtigt wurde. Um die individuelle Kompetenzentwicklung speziell dieser Lehrenden kurz zu skizzieren, sei auf die Studien von Bonnes und Hochholdinger (2016) und Wißhak et al. (2020) verwiesen. Hiernach gestalten sich Bildungsbiografien und Kompetenzerwerb, ähnlich wie im gesamten Weiterbildungsbereich, heterogen und wenig formalisiert. Trotz eines hohen Akademisierungsanteils (80 %) besitzen nur etwa 16 % der Lehrenden einen pädagogischen Hochschulabschluss, dafür sind sie im Erwerb non-formaler Zertifikate sehr aktiv (86 %). Vor allem besuchen sie sogenannte Trainerausbildungen, in welchen sie methodisch-didaktische Handlungskompetenzen erwerben (Wißhak und Hochholdinger 2015).

### Auswirkungen der Pandemie auf Lehrende in der Weiterbildung

Wie sich die Pandemiesituation für Lehrende in der Weiterbildung darstellt, wurde bisher nur in wenigen Erhebungen betrachtet. Christ und Koscheck (2021) berichten Ergebnisse der wbmonitor-Umfrage, in der insgesamt 1933 Weiterbildungseinrichtungen zu den Folgen der Pandemie befragt wurden. Demzufolge mussten die Einrichtungen zunächst 77 % der geplanten Kurse kurzfristig absagen oder verschieben. Längerfristig konnten sie Veranstaltungen entweder virtuell durchführen oder in Präsenz mit reduzierter Teilnehmendenzahl anbieten. Dies führte zu Umsatzeinbußen, weshalb nur 29 % der Befragten ihre wirtschaftliche Lage als positiv einschätzten, knapp halb so viele wie im Vorjahr. Der Anteil derer, die die wirtschaftliche Lage als negativ einschätzten, stieg von 12 % auf 42 %.

Aus der wbmonitor-Umfrage geht weiterhin hervor, dass während der ersten Öffnungsphase im Sommer 2020 etwa ein Fünftel der Veranstaltungen erstmalig in Online-Formaten durchgeführt wurde. Die Corona-Pandemie ging also mit einem Digitalisierungsschub einher (Kohl und Denzl 2020), während die Nutzung digitaler Technologien wie auch die entsprechenden Voraussetzungen der Lehrenden für digitale Lernangebote vor der Pandemie noch als ausbaufähig eingeschätzt wurden (Autorengruppe Bildungsberichterstattung 2021).

Zusammenfassend war für den Weiterbildungsbereich die Zeit während und nach der ersten Pandemiewelle von plötzlichen, einschneidenden Änderungen durch organisatorische und methodisch-didaktische Umstellungen sowie finanzielle Einbußen und Unsicherheit geprägt. Dies gilt auch für die Lehrenden. Ein ähnliches Bild speziell für den Bereich der betrieblichen Weiterbildung liefert eine Verbandsumfrage des Bundesverbands betriebliche Weiterbildung (Wuppertaler Kreis e. V. 2020).

Wie Lehrende, Programmverantwortliche und Leitende in der Weiterbildung die Veränderungen während und nach der ersten Pandemiewelle erlebten und insbesondere, wie sie damit umgingen, betrachtet eine qualitative Interviewstudie von Grotlüschen und Weis (2021). Dabei zeigte sich, dass die neue Situation zunächst als unklar und belastend eingeschätzt wurde, dies aber eine größere Offenheit für Veränderungen und Erprobungen sowie eine höhere Fehlertoleranz mit sich brachte, auch von Seiten der Zielgruppen. Die Entwicklung digitaler Formate wurde schnell als notwendig erkannt und akzeptiert, wobei die physische Distanz, vor allem für aktivierende und interaktive Trainingselemente problematisiert wurde. Dabei erwiesen sich ein intensiver Austausch mit anderen Lehrenden und Verantwortlichen sowie die eigene Weiterqualifizierung als hilfreich. Außerdem entdeckten die Interviewten neue Potenziale der digitalen Formate, etwa, dass sie dadurch bisher wenig erschlossene Zielgruppen erreichen konnten und räumlich unabhängiger waren. Im Rückblick empfanden die Befragten den Umgang mit eigenen Emotionen, die mit den Veränderungen und der Unsicherheit einhergingen, als herausfordernd.

### Professionelle Handlungskompetenzen von Lehrenden in der berufsbezogenen Weiterbildung

Die oben aufgeführten Studien zur Pandemie stellen heraus, dass die Lehrenden mit einschneidenden Veränderungen konfrontiert wurden. Dabei mussten sie ihr methodisch-didaktisches Handlungsrepertoire im Bereich der Digitalisierung rasch und umfassend erweitern. Auch mussten sie die größtenteils herausfordernden und belastenden Veränderungen auf emotionaler und sachlicher Ebene bewältigen.

**Figure 1:**
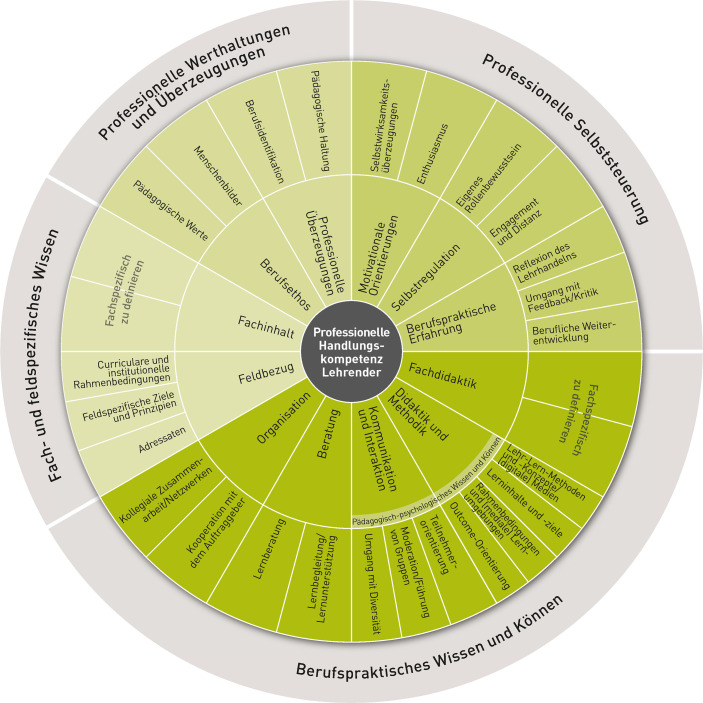

Sowohl die didaktischen Anforderungen als auch die Bewältigung der beruflichen Belastungen adressieren professionelle Handlungskompetenzen, die Lehrende in der Weiterbildung benötigen. Solche Handlungskompetenzen konkretisiert das GRETA-Kompetenzmodell (Strauch et al. 2019). Das Modell wurde theorie- und evidenzbasiert entwickelt unter Rekurs auf einschlägige Kompetenzmodelle und Befunde der empirischen Unterrichts- und Weiterbildungsforschung, wobei es für das Feld der Erwachsenen- und Weiterbildung spezifiziert wurde. Auf der Grundlage dieses Modells lassen sich non-formal oder informell erworbene Kompetenzen von Lehrenden anerkennen und Entwicklungsbedarfe ermitteln. Zugleich lässt es sich als ein breites und übergreifend angelegtes Modell nutzen, um theoriebasiert spezifische Kompetenzanforderungen zu kategorisieren und zu beschreiben, die sich aus der Situation der Corona-Pandemie ergeben. Zu diesem Zweck soll das GRETA-Kompetenzmodell in der vorliegenden Arbeit herangezogen werden. Das Modell unterscheidet zunächst vier übergreifende Kompetenzaspekte: berufspraktisches Wissen und Können, fach- und feldspezifisches Wissen, professionelle Selbststeuerung sowie professionelle Werthaltungen und Überzeugungen. Für die besonderen Anforderungen, die sich aus der Corona-Pandemie ergaben, sind vor allem Facetten des berufspraktischen Wissens und Könnens sowie der professionellen Selbststeuerung relevant (Abb. 1).

Die Digitalisierung von Lehr-Lernprozessen lässt neue Anforderungen hinsichtlich des pädagogisch-psychologischen Wissens erwarten. Dazu zählen im GRETA-Kompetenzmodell zunächst didaktische und methodische Gesichtspunkte. Auch müssen alternative Möglichkeiten für Kommunikation und Interaktionen gefunden werden, wenn diese nicht mehr oder nur begrenzt präsenzförmig möglich sind. Solche mediendidaktischen Kompetenzen adressieren auch Schmidt-Hertha et al. (2020) in ihrem Modell der medienpädagogischen Kompetenz. In der beruflichen und betrieblichen Weiterbildung benötigen Lehrende zudem Fähigkeiten, um den Lerntransfer der Teilnehmenden in ihren Arbeitsalltag zu fördern (Wißhak und Hochholdinger 2018).

Über das berufspraktische Wissen und Können hinaus sind Selbstregulation und Bewältigung mit Blick auf die pandemiebedingten Veränderungen und Unsicherheiten wichtig, welche die Lehrenden überwiegend als belastend erlebten. Dies betrifft überwiegend die Kompetenzbereiche Selbstregulation und motivationale Orientierungen des GRETA-Modells. Der Kompetenzbereich der Selbstregulation umfasst sowohl das Rollenbewusstsein als auch den verantwortungsvollen Umgang mit persönlichen Ressourcen im Spannungsfeld zwischen Engagement und Distanz. Facetten der motivationalen Orientierung betreffen den Enthusiasmus der Lehrenden sowie ihre Selbstwirksamkeitsüberzeugungen.

Aus psychologischer Sicht können belastende Situationen einerseits auf der praktischen Ebene tatkräftig bewältigt werden, also durch problemorientierte Coping-Strategien, andererseits auf der gefühlsmäßigen Ebene, durch emotionsorientierte Coping-Strategien (Lazarus und Folkman 1984). Solche unterschiedlichen Strategien sind auch in der Studie von Grotlüschen und Weis (2021) angedeutet.

Vor dem Hintergrund einschneidender und belastender Umbrüche durch die Corona-Pandemie, sowohl bezüglich der eigenen Existenzgrundlage als auch bezüglich der Digitalisierung, soll unsere Studie drei zentrale Forschungsfragen adressieren, die sich auseinander ergeben:

1. Welche unmittelbaren Auswirkungen hat die Corona-Pandemie auf die berufliche Situation der Lehrenden?
2. Welche Anforderungen stellen die neuen Gegebenheiten an ihre pädagogisch-psychologischen Kompetenzen insbesondere im Bereich der Digitalisierung?
3. Welche Kompetenzen im Bereich der Selbstregulation und der Bewältigung sind nötig, um mit den Veränderungen der beruflichen Situation und der benötigten didaktischen Kompetenzen umzugehen?

## Methode

Für die Studie wurde textbasiertes, überwiegend schriftliches Material erhoben und qualitativ ausgewertet, worin Lehrende sich zu den Forschungsfragen äußerten.

### Studienteilnehmende und Datenquellen

Insgesamt beteiligten sich 23 Personen oder Teams. Drei Beiträge wurden nicht ausgewertet, da sie nicht von Lehrenden aus der Weiterbildung stammten. Somit wurden die Beiträge von 20 Lehrenden bzw. Teams analysiert. Mit drei der 20 Lehrenden wurden vertiefende Interviews geführt.

Aufgrund der Erhebungsmethode liegen nur wenige soziodemografische Informationen vor. Elf Lehrende waren weiblich und 6 waren männlich. Drei Beiträge wurden von mehreren Personen gemeinsam verfasst. Neben Trainerin bzw. Trainer gaben die Befragten weitere Berufsbezeichnungen an, wie Coach, Dozentin bzw. Dozent, Speaker, Beraterin bzw. Berater, Unternehmerin bzw. Unternehmer, Digital Learning Expert, Change Managerin und Manager, Consultant, Mediatorin bzw. Mediator und Personal- und Organisationsentwicklerin bzw. -entwickler. Im Mittel besaßen sie eine Berufserfahrung im Trainerberuf von 11,34 Jahren. Fast alle Befragten arbeiteten in Deutschland, bis auf eine Person, die überwiegend in der Schweiz tätig war.

Das ausgewertete Datenmaterial bestand aus 22 Quellen: 16 Texten (Blogbeiträgen), einem Video, einer Audiodatei, einer Zeichnung, die auch Text enthält, sowie drei Interviews, die ebenfalls als Videos vorliegen. Die Video- und Audiodateien wurden für die Auswertung zunächst transkribiert und anonymisiert. Zwei der Interviews dauerten jeweils 15 min, das dritte dauerte 49 min. Die analysierten Dokumente umfassen insgesamt 30.629 Wörter (*M* = 1423, Min = 80, Max = 7406).

### Teilnehmendenrekrutierung

Für die Datengewinnung riefen wir in Zusammenarbeit mit dem Weiterbildungsanbieter Dr. Sammet & Wolf Lehrende zur Teilnahme an einer sogenannten Webparade auf. Hierbei wurden die Teilnehmenden gebeten, in einer von ihnen selbst gewählten medialen Form auf vorgegebene Fragen bezüglich der Corona-Pandemie zu antworten. Der Aufruf wurde über die Internetseite des Weiterbildungsanbieters sowie über Karrierenetzwerke wie LinkedIn und Xing kommuniziert. Die Teilnahme an der Webparade war freiwillig und wurde nicht vergütet. Jedoch wurden die Lehrenden darauf hingewiesen, dass eine Teilnahme an der Webparade ihre Sichtbarkeit erhöhen und somit als Marketingmaßnahme dienen kann. Drei Lehrende konnten für ein Interview gewonnen werden. Damit wurde eine Gelegenheitsstichprobe gezogen. Alle Teilnehmenden wurden darüber informiert, dass die Beiträge wissenschaftlich ausgewertet und veröffentlicht würden.

### Datenerhebung

Die Erhebung fand im August und September des Jahres 2020 statt, also zwischen der ersten und zweiten Pandemiewelle. Die teilnehmenden Lehrenden orientierten sich an den in der Webparade gestellten offenen Fragen, bestimmten die mediale Aufbereitung aber selbst. In den Interviews wurden die gleichen Fragen gestellt. Dort konnte die Interviewerin jedoch Fragen nach Bedarf paraphrasieren oder gezielte Nachfragen stellen.

Folgende Fragen wurden in der Webparade und den Interviews an die Lehrenden gestellt:

1. Was verändert sich für Trainerinnen und Trainer durch COVID-19?
2. Was sind die größten Herausforderungen für Trainerinnen und Trainer im Umgang mit diesen Veränderungen?
3. Was ist hilfreich im Umgang mit diesen Herausforderungen?
4. Welcher Nutzen ergibt sich aus den Veränderungen für Organisationen und Trainerinnen und Trainer?
5. Was sollten Trainerinnen und Trainer können, um weiterhin erfolgreich zu sein?

Alle Beiträge wurden durch die Lehrenden im Internet bereitgestellt und dort von den Forschenden heruntergeladen. Die videografierten Interviews wurden ebenfalls – mit der schriftlichen Einwilligung der Lehrenden – im Internet veröffentlicht. Nach Ende des Erhebungszeitraums wurden die Berufe der Teilnehmenden überprüft und die drei Beiträge, die nicht von Trainerinnen und Trainern stammten, von der Analyse ausgeschlossen.

### Analysen

Ziel der qualitativen Studie war es, aus dem Material möglichst umfassende Antworten auf die Forschungsfragen zu erhalten. Das Textmaterial wurde mit der qualitativen Inhaltsanalyse nach Mayring (2014) ausgewertet. Diese Methode ermöglicht durch eine Zuordnung zu Kategorien zunächst eine gebündelte inhaltliche Interpretation von Aussagen und darauf aufbauend weiterhin eine quantitative Auswertung der Häufigkeiten, mit der Themenbereiche genannt werden. Das zu diesem Zweck entwickelte Kategoriensystem enthält deduktive Kategorien, die pädagogische und psychologische Konzepte aus der oben dargestellten Forschungslage beschreiben (Strauch et al. 2019; Schmidt-Hertha et al. 2020). Hinzu kommen induktive Kategorien, die aus dem Material entwickelt wurden. Die insgesamt 22 Kategorien sind den drei oben genannten Forschungsfragen nach unmittelbaren Auswirkungen, didaktischen Anforderungen und Bewältigungsstrategien zugeordnet. Anforderungen und Kompetenzen werden in der Auswertung nicht systematisch unterschieden, da die Lehrenden in ihren Beiträgen meist keinen Unterschied machten. Um eine hohe Trennschärfe zwischen den Kategorien zu gewährleisten, wurden sie in iterativen Schritten erprobt und optimiert. Das Codierschema enthält neben den 22 Kategorien und deren Definitionen jeweils mindestens ein Ankerbeispiel in Form eines prototypischen Zitats aus dem Material sowie einige Regeln zur Reichweite und Abgrenzung der Kategorien (Mayring 2014). Die Kategorien, ihre Definitionen und Codierhäufigkeiten sind in Tab. 1 aufgeführt.

**Table 1:** 

Kategorie	Definition	Codierhäufigkeit
1 Auswirkungen der Corona-Pandemie	Unmittelbare berufliche Folgen: Was hat sich (nicht) verändert?	–
1.1 Auftragslage und finanzielle Situation	Unmittelbare Auswirkungen der Pandemie auf die Auftragslage und finanzielle Lage der Lehrenden sowie auf ihre Zusammenarbeit mit Unternehmen	44
1.2 Corona als Digitalisierungskatalysator	Aussagen, die sich darauf beziehen, dass die Pandemie die Digitalisierung ausgelöst oder beschleunigt hat (im Hinblick auf die gesamte Gesellschaft, die Lehre oder Geschäftsmodelle)	41
1.3 Verunsicherung	Lehrende sind von der Corona-Krise und der Digitalisierung zunächst verunsichert	13
1.4 Finanzielle und zeitliche Vorteile digitaler Lehre	Finanzielle, zeitliche und logistische Vorteile für Lehrende, Teilnehmende und Unternehmen durch digitale Lehre	17
1.5 Verbesserte Work-Life-Balance	Positive, faktische oder mögliche Auswirkungen auf die Vereinbarkeit von Beruf und Privatleben	6
1.6 Unverändert benötigte Kompetenzen	Kompetenzen der Lehrenden, die vor der Pandemie wichtig waren und es auch weiter sein werden	11
2 Didaktische Anforderungen und Kompetenzen	Methodisch-didaktische und kommunikative Anforderungen, die sich durch die Umstellung auf digitale Lehr-Lern-Angebote stellen und Kompetenzen, die sie benötigen, um den Anforderungen gerecht zu werden	–
2.1 Präsenztrainings nicht 1:1 digitalisierbar	Lehr-Lernangebote, die vorher in Präsenzform stattfanden, können nicht unverändert digital stattfinden: Es bedarf einer Anpassung auf verschiedenen Ebenen (z. B. Format, Didaktik)	15
2.2 Digitale mediendidaktische Kompetenz	Lehrende benötigen digitales mediendidaktisches und -psychologisches Wissen, Kenntnisse über digitale Lehr‑/Lerntechnologien	35
2.3 Primat der Lernziele und Didaktik vor der medialen Gestaltung	Inhalte und didaktische Überlegungen sollen die mediale Gestaltung bedingen, nicht umgekehrt	5
2.4 Lernenden- und Teilnehmendenorientierung	Lehr-Lernangebote sollen nun noch individueller und flexibler an die Bedarfe der Lernenden und der auftraggebenden Unternehmen/Kunden angepasst werden	16
2.5 Problem der physischen Distanz	Durch die physische Distanz und die Wahrnehmung über den Computerbildschirm können einige Methoden nicht eingesetzt werden und manche körpersprachliche Signale werden nicht wahrgenommen; die Herstellung von gefühlter Nähe wird schwieriger	24
2.6 Moderation/Führung von (Lern)Gruppen	Moderation und kommunikative Steuerung von Gruppenprozessen verändern sich	5
2.7 Andere Formate und Taktung digitaler Lerneinheiten	Digitales Lehren und Lernen erfolgt in anderen Formaten; dies bezieht sich nicht nur auf den Ort und das Medium, sondern auf die Implikationen und Möglichkeiten, die dadurch für den gesamten Lernprozess entstehen	24
2.8 Outcomeorientierung und Transfer	Lehrende sollen den Lernerfolg, die langfristige Anwendung des Gelernten im Arbeitsalltag sowie die erwünschten Resultate auf Unternehmensebene fördern	16
2.9 Notwendigkeit und Möglichkeiten der eigenen Weiterbildung	Lehrende sollen sich weiterbilden, um den neuen Anforderungen gerecht zu werden. Fortbildungen und Zertifikate für Lehrende, aber auch informelle Weiterbildungsmöglichkeiten	13
3 Selbstregulation und Bewältigung	Fähigkeiten der Selbstregulation und Bewältigung sowie motivationale Orientierungen, die Lehrende im Umgang mit den Auswirkungen der Pandemie benötigen sowie Strategien, von denen sie berichten oder die sie empfehlen	–
3.1 Umgang mit Veränderung, Komplexität und Unsicherheit	Lehrende benötigen die Fähigkeit, mit Veränderungen, Komplexität und Unsicherheiten umzugehen	11
3.2 Rollenbewusstsein	Lehrende reflektieren ihre Rolle und deren Veränderung	24
3.3 Deutung der Pandemie als Lerngelegenheit und Anlass zur Reflexion und Neupositionierung	Lehrende nehmen die Pandemie oder die Digitalisierung als Lerngelegenheit und Anlass zur Selbstreflexion und Neupositionierung war	54
3.4 Netzwerken	Soziale (berufliche) Ressourcen nutzen: Lehrende vernetzen sich mit anderen Lehrenden, nehmen und bieten Hilfe an	24
3.5 Pragmatismus	Distanzierungsfähigkeit/Pragmatismus im Umgang mit den Herausforderungen	10
3.6 Selbstwirksamkeit und sich auf die eigenen Stärken besinnen	Lehrende benötigen eine hohe Selbstwirksamkeitserwartung und Selbstvertrauen für den Umgang mit den Herausforderungen und sollen sich auf ihre Stärken besinnen	6
3.7 Achtsamkeit und Selbstfürsorge	Emotionsorientierte Bewältigung mit Blick auf das eigene Wohlbefinden	6

Alle Beiträge sowie das Codierschema wurden in das Analyseprogramm MAXQDA (VERBI Software 2020) eingepflegt und darin ausgewertet. Berücksichtigt wurden alle Textstellen, die inhaltlich relevant für die Beantwortung der drei Fragestellungen waren. Als Codiereinheit, also als kleinstmögliche codierbare Textstelle, wurden ein Satz oder bei Aufzählungen ein Stichpunkt festgelegt. Die analysierte Kontexteinheit bestand in einem Text oder einer kommentierten Zeichnung. Die Auswertungseinheit stellte die Gesamtheit der 22 ausgewerteten Dokumente dar. Es wurden 420 Textstellen codiert.

Für die Berechnung der Interraterübereinstimmung wurde eine wissenschaftliche Hilfskraft mit dem Codierleitfaden vertraut gemacht. Zunächst wurden fünf Beiträge von der Hilfskraft und einer der Autorinnen getrennt codiert, um jeweils im Anschluss etwaige Abweichungen zu besprechen. Der für weitere fünf codierte Texte berechnete, zufallsbereinigte Übereinstimmungskoeffizient Kappa nach Brennan und Prediger (1981) liegt bei 0,75. Er ist als ausreichend anzusehen (Cohen 1960), sodass das übrige Material von einer der Autorinnen codiert werden konnte.

## Ergebnisse

In diesem Beitrag wurden Ausführungen von Lehrenden zu Folgen der Corona-Pandemie hinsichtlich spezifischer Kompetenzanforderungen und Bewältigungsstrategien ausgewertet. Dabei hängen die drei Forschungsfragen logisch zusammen: Die unmittelbaren Auswirkungen der Corona-Pandemie erfordern eine methodisch-didaktische Umstellung auf digitale Lehr-Lernangebote und die dafür nötigen Kompetenzen. Angesichts von beidem benötigen die Lehrenden selbstregulative Kompetenzen und Bewältigungsstrategien.

Für die erste Forschungsfrage zu von Lehrenden genannten unmittelbaren Auswirkungen der Corona-Pandemie auf ihre berufliche Situation wurden insgesamt sechs Kategorien identifiziert:

Die *Auftragslage und finanzielle Situation* wurde insgesamt 44 Mal in 17 Beiträgen codiert. Dabei berichteten die Lehrenden Sparmaßnahmen der Unternehmen und den dadurch bedingten Wegfall von Aufträgen (z. B. P.2, P.3, P.4, P.6, P.7). Zudem charakterisierten sie die Auftragslage als unberechenbar und wiesen darauf hin, dass sich die Kommunikation mit Unternehmen generell verändert habe (z. B. P.6, P.7). Zu Beginn der Pandemie fand zunächst wenig Austausch statt. Es kamen neue Auftraggeber hinzu, während andere wegfielen (P.2). Manche Kundinnen und Kunden müsse man erst von den Vorteilen digitaler Lehre überzeugen (P.13). Manche Lehrende nahmen auch einen erhöhten Weiterbildungsbedarf wahr, bei gleichzeitiger Verschiebung der Verantwortung für die eigene Weiterbildung auf die Teilnehmenden, da die Unternehmen an Weiterbildung sparten (P.6, P.11).

Bezüglich der Aussage, dass die Corona-Pandemie als *Digitalisierungskatalysator *fungiere, waren sich 17 Lehrende in 41 Textstellen einig. „Bedingt durch Stornierungen und vermutlich auch mittelfristig rückläufige Auftragslage, muss das lange aufgeschobene Thema „Digitale Angebote“ ganz oben auf die To-Do-Liste. Entschuldigungen, Bequemlichkeit und Berührungsängste sind im Angesicht der Notwendigkeit verblasst. Der Sprung ins kalte digitale Wasser muss sein – Augen zu und durch!“ (P.2, 4).

Dass die Corona-Pandemie mit einer *Verunsicherung* der Lehrenden einherginge, äußerten neun Personen in 13 Textstellen, „[…] Trainer*innen fühlen sich unsicher und verletzlich, leiden unter mangelndem Selbstbewusstsein und fühlen sich zurückversetzt in die Anfangszeit ihrer Trainerkarriere, als sie sich ohne Erfahrung und Referenzen ihre Reputation mühsam erarbeiten mussten“ […] (P.9, 20). Während erfahrene Lehrende sich im Seminarraum sicher fühlten, ginge diese Sicherheit im digitalen Raum zunächst verloren. Hinzu komme eine Verunsicherung aufgrund der pandemiebedingten prekären finanziellen Lage vieler Lehrenden (P.13).

Auch positive Effekte der Corona-Pandemie wurden von neun Lehrenden in 17 codierten Textsegmenten berichtet. Demzufolge habe die digitale Lehre durch wegfallende Reisen *finanzielle und zeitliche Vorteile* für Lehrende, Teilnehmende und Unternehmen (z. B. P.2, P.3, P.7).

Eine *bessere Work-Life-Balance* ermöglicht die Umstellung auf digitale Lehre für fünf Befragte. Die sechs codierten Segmente beziehen sich auf mehr Zeit mit Familie und Freunden (P.2, P.18), die Gesundheit und eine bessere Lebensqualität (P.9) sowie die Möglichkeit, seinen Arbeitsort frei zu wählen (P.11).

Auch *unverändert benötigte Kompetenzen* nannten sieben Lehrende in elf codierten Textstellen. Aufgeführt wurden das Fachwissen (P.3, P.12, P.13), die berufspraktische Erfahrung (P.9, P.12), pädagogische Werte (P.9) sowie die pädagogische Haltung (P.21, P.22). „Als allererstes fällt mir da natürlich die Kompetenz im eigenen Thema ein sowie die Erfahrung als Trainer, egal ob als Präsenz- oder Online-Trainer. Letztlich geht es immer darum, mit einer Gruppe von Menschen eine optimale Lernumgebung zu erzeugen und zu gestalten“ (P.3, 22).

Im Rahmen der zweiten Forschungsfrage zu didaktischen bzw. pädagogisch-psychologischen Anforderungen und Kompetenzen im Bereich der Digitalisierung wurden neun Kategorien identifiziert:

Dass *präsenzförmige Lehr-Lernangebote nicht 1:1 digitalisiert* werden können, äußerten elf Lehrende in 15 Textstellen: „Ein Präsenztraining kannst du nicht einfach 1:1 online durchführen“ (P.13, 47). Drei Lehrenden berichteten außerdem, dass die Vorbereitung von Online-Lehre aufwendiger sei und mehr Zeit benötige (P.2, P.8, P.21).

Die Notwendigkeit einer *digitalen mediendidaktischen Kompetenz* betonten 13 Lehrende in 35 Textsegmenten. Die codierten Inhalte umfassen überwiegend digitales mediendidaktisches und -psychologisches Wissen sowie anwendungsbezogene Kenntnisse über digitale Lehr-Lerntechnologien, etwa der Umgang mit Tools der digitalen Lehre und die Unterstützung der Lernenden bei technischen Problemen. „Sondern jetzt ist die Frage, welche Online-Tools nehme ich? Nehme ich ein Ural, nehme ich ein Miro-Board? Switche ich da wirklich das Medium oder nehme ich das Whiteboard, was mir mein ähm Zoom zum Beispiel anbietet […]“ (P.11, 67). „Damit du in Zukunft online genauso locker wie in einem Seminarraum improvisieren kannst, musst du dich weiter mit der Technik auseinandersetzen und den Umgang mit den verschiedenen Tools perfektionieren“ (P.13, 43). „Mit anderen Worten, du brauchst einen erweiterten methodisch-didaktischen und technischen Werkzeugkoffer, um digitale Lernräume sinnvoll zu gestalten“ (P.13, 55).

Auf das *Primat der Lernziele und Didaktik* gegenüber der medialen Gestaltung wies eine Person fünfmal hin. Demzufolge sollten digitale Technik und Tools lediglich Werkzeuge sein, um die Lernziele zu erreichen. „Du sollst kein Sklave der Technik werden, vielmehr soll sie dich in deinen Zielen unterstützen. Deshalb frage dich immer erst, was du eigentlich erreichen willst“ (P.13, 76).

Die *Lerner‑/Teilnehmerorientierung* wurde von zehn Lehrenden in 16 codierten Segmenten sowohl als Anforderung als auch Chance wahrgenommen. So seien Lehr-Lernangebote zukünftig individueller und flexibler an die Weiterbildungsbedarfe der Teilnehmenden und der Unternehmen anzupassen. „[…] Man spricht auch vom NEW LEARNING, in dessen Fokus der Lernende selbst und seine Bedürfnisse und Lernziele stehen“ (P.16, 48).

Die *physische Distanz* in der Online-Lehre bewerteten die Lehrenden als problematisch (12 Befragte, 24 Segmente). Viele Methoden seien online nicht oder schwieriger umzusetzen (z. B. P.15, P.22). Gleichzeitig seien körpersprachliche Signale nicht oder schwieriger wahrnehmbar (P.2, P.15). Überdies fällt der informelle Austausch in den Pausen weg (P.13, P.15). Persönliche Begegnungen und Beziehungen seien schwieriger herzustellen (P.16). Manche Lehrende kamen daher zu dem Schluss, dass bestimmte, weiche, Themen nicht online gelehrt werden können. „Bestimmte Kompetenzen wird man allerdings auch in Zukunft nur im Präsenztraining aufbauen können, weil es da einfach das eigene Erleben bzw. ein direktes Gegenüber braucht. So könnte ich mir ein Training zu Gesprächsführung oder gar eine Coaching-Ausbildung nicht als reines Online-Training vorstellen“ (P.3, 19). Andere Befragte äußerten sich zuversichtlicher und stellten lediglich fest, dass in solchen Trainings besondere Anstrengungen nötig seien, um auch in digitalen Räumen eine gewisse Nähe herzustellen.

Auf veränderte Anforderungen bei der *Moderation* in digitalen Lehrveranstaltungen gingen drei Befragte in fünf Textstellen ein. Digitale Lehrveranstaltungen müssten straffer moderiert werden. „Du bist als Moderator viel mehr gefragt: Du musst deine Trainings noch besser strukturieren und viel stärker steuern, damit du deine Teilnehmenden immer wieder aktivieren kannst“ (P.13, 50). Die Lehrenden sahen dabei v. a. die Herausforderung, die Gruppe der Lernenden im Blick zu behalten. Person 11 schlug in dem Zusammenhang vor, entweder eine Trainerkollegin bzw. einen Trainerkollegen oder eine teilnehmende Person für die Co-Moderation einzubinden.

Mögliche *Formate und Taktung digitaler Lehre* wurden von neun Befragten in 24 codierten Segmenten überwiegend positiv bewertet. In der räumlichen und zeitlichen Flexibilität sehen sie Vorteile für den gesamten Lernprozess, die entsprechend genutzt werden sollten. Konkret berichten sie, dass digitale Lehre in kleineren Einheiten, dafür aber über einen längeren Zeitraum angeboten werden kann (P.2, P.11, P.13). Auch auf die Vorzüge von Blended Learning, also einer Kombination aus digitalen und Präsenzanteilen wurde häufiger hingewiesen (z. B. P.13). Zudem lassen sich sogenannte Follow-Up Maßnahmen, z. B. einige Monate nach der eigentlichen Intervention, digital mit wenig Aufwand umsetzen (P.3, P.13). Aus einem sich aus einem Training ergebenden individuellen Bedarf kann außerdem unkompliziert ein zusätzliches Coaching entstehen. Solche individuellen Angebote sollten Lehrende laut Person 11 anbieten können. Gleichzeitig lassen sich durch digitale Weiterbildung auch weltweit verstreute Mitarbeitende eines Unternehmens einbeziehen (P.9).

Auf den *Transfer*, also die Umsetzung des Gelernten am Arbeitsplatz, bezogen sich sechs Lehrende in 16 Textstellen. Die Lehrenden sehen hier überwiegend Aspekte digitalen Lernens, die den Transfer erleichtern, z. B. das Erproben des Gelernten zwischen mehreren Online-Treffen (P.2). Es werden aber auch neue Hürden wahrgenommen. Bspw. wird befürchtet, dass die Umsetzung allein im Homeoffice weniger leicht gelingt, als gemeinsam am Arbeitsplatz (P.11). Person 11 weist auf die Transfervoraussetzung hin, dass sich Lehrende mit den auftraggebenden Unternehmen abstimmen, um Lern- und Entwicklungsprozesse gemeinsam zu planen und umzusetzen.

Die *Notwendigkeit und verschiedene Möglichkeiten der Weiterqualifizierung* sprachen elf Lehrende in 13 Segmenten an und empfahlen, eine Online-Trainer-Ausbildung zu machen (P.3, P.6, P.8, P.9, P.14, P.16, P.19). Auch auf informelle Weiterbildungsmöglichkeiten wie Literatur, Blogs und kollegialen Austausch wurde hingewiesen (P.7, P.21). Person 18 merkte an, dass sich Lehrende zunächst überlegen sollten, was sie für ihre individuelle Weiterentwicklung benötigten, statt Zertifikate anzuhäufen.

Im Sinne der dritten Forschungsfrage waren Äußerungen zu Fähigkeiten der Selbstregulation und Bewältigungsstrategien sieben Kategorien zuzuordnen:

Dass Lehrende generelle *Fähigkeiten im Umgang mit Veränderung, Komplexität und Unsicherheit* benötigen, gaben sechs Befragte in elf Textstellen an. Dabei zeige sich in der Krise auch, ob Lehrende derartige Kompetenzen, die sie teilweise in ihren Trainings und Coachings zu vermitteln versuchen, auch selbst beherrschen (P.3).

Zu dem sich verändernden *Rollenbewusstsein* der Lehrenden äußerten sich zehn Personen in 24 codierten Segmenten. Die eigene Rolle müsse neu definiert und auch die Rollen aller anderen Beteiligten überdacht werden (P.2). Die Befragten waren dabei durchaus selbstkritisch: „Die Einzelkämpferhaltung und den Standesdünkel ablegen. In manchen Fällen auch verbunden mit einer gewissen Arroganz und überhöhter Selbstbewertung, sei es der eigenen Person oder der Fachkompetenz gegenüber“ (P.2, 15). In der neuen Rolle sind die Lehrenden gleichzeitig auch Lernende. „Konkret bedeutet dies aktuell, die eigene Rolle vom Wissensvermittler zum Agilen Lerncoach weiterzuentwickeln und vo[m] Berater zum Begleiter der agilen Prozessentwicklung zu werden“ (P.6, 53).

Die Krise als *Lerngelegenheit und Anlass zur Reflexion und Neupositionierung* anzusehen, empfahlen 14 Personen in 54 Codiereinheiten. Die erzwungene Veränderung wird damit zur positiven Entwicklungsmöglichkeit umgedeutet, die sogar Spaß machen kann. „Auch die Aneignung der technischen Möglichkeiten und die Anpassung der Inhalte auf das Medium „online“ entsprechend als Zugewinn zu empfinden und nicht als lästiges Übel“ (P.2, 9). Auch die anfängliche freie Zeit, die durch stornierte Weiterbildungen entstand, kann als Chance umgedeutet werden, die eigenen Angebote umzustellen bzw. Themen und Veränderungen anzugehen, zu denen man sonst nicht kam (P.6). „Hm… ah, also der große Nutzen und die große Chance ist natürlich, sich selber und sein Business nochmal zu reflektieren. Ist das weiter das, wie ich es machen wollte oder ist es ein guter Anlass, auch selber Produkte umzustrukturieren, umzustricken auf den Kunden“ (P.11, 204f). Zu dieser Kategorie der Selbstregulation gehören neben Lernbereitschaft eine generelle Offenheit für Neues und ein iteratives Vorgehen bei der Umstrukturierung von Lehr-Lernangeboten (z. B. P.13).

Das *Netzwerken* ist eine weitere, häufig genannte Bewältigungsstrategie. 14 Lehrende empfahlen in 24 Einheiten, mit Kolleginnen und Kollegen zusammenzuarbeiten, Hilfe anzubieten und anzunehmen, Lerngruppen zu bilden, aus dem Erfahrungsschatz anderer zu schöpfen, neue Formate und Methoden aneinander auszuprobieren und sich gegenseitig Feedback zu geben (P.2, P.14, P.12, P.23). Auch Feedback von Teilnehmenden und Kundinnen und Kunden wird als wichtige Ressource angesehen (P.4, P.21, P.23). Entgegen dem häufig verwendeten Begriff des „social distancing“ dürfe physische Distanz nicht zu sozialer Distanz führen (P.22, 33).

Sieben Personen waren sich in zehn Segmenten einig, dass *Pragmatismus* in Zeiten der Krise dem Perfektionismus vorzuziehen sei (z. B. P.4, P.9). Sie empfahlen, nachsichtig mit sich zu sein und auch den Kundinnen und Kunden gegenüber zu kommunizieren, was möglich sei und was nicht (P.2).

Darauf, dass in Zeiten der Corona-Pandemie die *Selbstwirksamkeitsüberzeugung* wichtig sei, und dass sie sich *auf ihre Stärken besinnen* sollten, wiesen vier Lehrende in sechs codierten Elementen hin. „Vertrauen in sich selbst und die eigenen Fähigkeiten“ (P.16, 30). Dies bezieht sich teilweise auf die Kategorie zur ersten Forschungsfrage *unverändert benötigte Kompetenzen*. „Und das sollten wir auch […] nutzen und unser Selbstbewusstsein damit auch stärken und sagen, das ist das, was nicht verloren geht, sondern das was wir nach wie vor … ähm einbringen und mitbringen für diese Trainings“ (P.9, 48).

Die Kategorie *Achtsamkeit und Selbstfürsorge* wurde in fünf Beiträgen und sechs codierten Elementen angesprochen. Die Lehrenden wiesen darauf hin, dass Rücksicht auf die eigenen Bedürfnisse und ein wertschätzender Umgang mit sich selbst wichtig sei (P.16). Sie berichteten emotionsorientierte Copingstrategien von der Meditation bis zum Einsatz eines Kuscheltiers als stellvertretendes Publikum bei Online-Veranstaltungen.

## Diskussion

Die Corona-Pandemie und die durch sie beschleunigte Digitalisierung des Weiterbildungssektors stellen unerwartete Ereignisse dar, die wissenschaftlich noch weiter aufgearbeitet werden müssen. Unsere Studie befasst sich mit den kurz- und mittelfristigen Folgen für den berufsbezogenen Weiterbildungsbereich, wie sie Lehrende im Spätsommer 2020 wahrnahmen. Ihre überwiegend textförmigen, im Internet veröffentlichten Beiträge wurden inhaltsanalytisch im Hinblick auf die pandemiebedingten Kompetenzanforderungen und deren Bewältigung ausgewertet.

Die Befunde fassen erlebte berufliche Veränderungen und neue Anforderungen an die pädagogisch-psychologischen Kompetenzen wie auch an die Selbstregulations- und Bewältigungsstrategien der Lehrenden systematisch zusammen.

Im Hinblick auf die erste Fragestellung äußerten die Lehrenden, dass der ohnehin anstehende Digitalisierungsprozess durch die Krise nur beschleunigt wurde und somit auch solche Trainerinnen und Trainer zwang, ihre Angebote umzustellen, die sich bisher gesträubt hatten. Zudem sei nicht nur die Lehre von diesem Wandel betroffen, sondern allgemeiner die Geschäftsmodelle und -prozesse in der betrieblichen Weiterbildung. Neben der anfänglichen Verunsicherung machten sie auch positive Auswirkungen aus, wie eine verbesserte Work-Life-Balance.

Bezüglich der zweiten Fragestellung nach pädagogisch-psychologischen Kompetenzen betonten die Lehrenden, dass das anfängliche, teils hektische Aneignen von neuen Tools und Lernplattformen im nächsten Schritt in eine didaktisch überlegte, ganzheitliche Konzeption digitaler Lehr-Lernangebote übergehen müsse. Hierfür benötigen Lehrende neben neuen mediendidaktischen Fähigkeiten und Qualifikationen auch bekannte Konzepte wie Teilnehmendenorientierung und Transferförderung. Neben vielen Vorteilen digitaler Lehre, wie der flexibleren Gestaltung von Trainingsformaten, fanden die Befragten vor allem den schwer zu erreichenden persönlichen Austausch problematisch.

Das pragmatische und oft autodidaktische Aneignen mediendidaktischer Kompetenzen bedingt durch die Digitalisierung wurde auch in anderen Studien berichtet, z. B. bei Bolten-Bühler (2021).

Bezüglich unserer dritten Frage nach der Selbstregulation und Bewältigung lässt sich zusammenfassen, dass die Lehrenden neben dem problemorientierten Angehen der Herausforderungen emotionsorientierte Copingstrategien berichteten. Vor allem das positive Umdeuten der Krise zu einer Lerngelegenheit wurde häufig berichtet. Mit Blick auf das GRETA-Kompetenzmodell könnte dies ein Weg sein, den beruflichen Enthusiasmus wiederzuerlangen. Ähnlich weist die Studie von Bolten-Bühler (2021) darauf hin, dass eine offene Einstellung gegenüber digitalen Medien sowie ein funktionaler medialer Habitus förderlich für die medienpädagogische Kompetenzentwicklung sein können.

Zudem empfahlen die Lehrenden, sich mit Gleichgesinnten auszutauschen und sich in Netzwerken zusammenzuschließen. Dies kann problemorientiert, z. B. durch Informationsaustausch, oder emotionsorientiert, durch gegenseitige emotionale Unterstützung, geschehen. Diese Bewältigungsstrategien erscheinen angemessen angesichts der Tatsache, dass Personen, die sich mit Trainings und Personalentwicklung beschäftigen, überdurchschnittlich extrovertierte und offene Persönlichkeitsmerkmale aufweisen. In einer entsprechenden Untersuchung von Sundstrom et al. (2016) hingen zudem Extraversion und Optimismus positiv mit der Arbeitszufriedenheit solcher Personen zusammen. Nittel und Seltrecht (2008) weisen außerdem darauf hin, dass soziale Netzwerke im Prozess der individuellen Professionalisierung von großer Wichtigkeit sind.

Auch die im GRETA-Kompetenzmodell enthaltene Facette Selbstwirksamkeitsüberzeugungen wurde angesprochen. Anstatt sich von den Herausforderungen verunsichern zu lassen, machten sich die Lehrenden bewusst, dass ihr vorhandenes Wissen und Können nach wie vor Relevanz besitzt. Gleichzeitig verwiesen die Befragten auf einen Rollenwandel weg von der Wissensvermittlung hin zur Lernbegleitung. Somit könnte die Digitalisierung den Rollenwandel noch beschleunigt haben, der laut Strauch et al. (2019) ohnehin stattfindet.

Hinsichtlich der Kompetenzfacette Engagement und Distanz des GRETA-Kompetenzmodells lässt sich folgern, dass die unter den Fragestellungen 1 und 2 diskutierten Anforderungen zunächst ein erhöhtes Engagement von vielen Lehrenden forderten. Entsprechend wird unter der Fragestellung 3 in den Kategorien Pragmatismus sowie Achtsamkeit und Selbstfürsorge empfohlen, sich zunächst vom Anspruch der Perfektion zu distanzieren und auf die eigenen Bedürfnisse und das eigene Wohlbefinden zu achten.

Unsere Ergebnisse lassen sich also gut in den theorie- und evidenzbasierten Forschungskontext und die einschlägigen Kompetenzmodelle für Lehrende in der Weiterbildung einbetten, insbesondere das GRETA-Kompetenzmodell (Strauch et al. 2019). Letzteres wird derzeit um digitale Kompetenzen ergänzt (Strauch und Alberti 2021). Dabei zeigt sich ebenso wie in unserer Studie, dass digitale Kompetenzen über das pädagogisch-psychologische Wissen hinausgehen und sich bspw. auch auf die Selbststeuerung beziehen. Unsere Befunde stehen außerdem im Einklang mit weiteren Befunden zu Auswirkungen der Corona-Pandemie auf die privatwirtschaftliche Erwachsenenbildung von Grotlüschen und Weis (2021). Auch dort wurden Austausch und Vernetzung, ein flexibles, iteratives, zielgruppenorientiertes Vorgehen, Agilität und Blended Learning als hilfreich und zukunftsweisend beschrieben. Bei aller Anpassungsbereitschaft wurde ebenso auf das Primat der Didaktik gegenüber dem Medium hingewiesen. Diese Einschätzung wird durch eine Meta-Analyse von Sitzmann et al. (2006) unterstützt. Dort wurde kein Unterschied zwischen webbasierten und Präsenztrainings im Hinblick auf den Lernerfolg gefunden, sofern dieselben Lehr-Lernprinzipien und -methoden verwendet wurden.

Neue Erkenntnisse liefert unsere Studie bezüglich möglicher Auswirkungen der Digitalisierung auf den Weiterbildungstransfer. So weisen manche Antworten zur zweiten Forschungsfrage auf mögliche Vorteile für die Transferförderung durch digitale Lehr-Lernangebote bzw. Blended Learning hin, da sie zeitlich flexibler sind als die meist blockförmigen Präsenzangebote. Digitale Lerneinheiten können zeitnah, flexibel und bedarfsgerecht zur Verfügung gestellt werden, ohne dass Lernende ihrer Arbeit fernbleiben müssen. Dadurch können Trainings über einen längeren Zeitraum berufs- und lernprozessbegleitend eingesetzt werden, was nachweislich die Transferwahrscheinlichkeit erhöhen kann (Salas et al. 2012). Dies lässt auf eine positive Entwicklung im Hinblick auf nachhaltige Weiterbildung hoffen.

Lehrende wiesen, wie bei Grotlüschen und Weis (2021) außerdem darauf hin, dass digitale Maßnahmen auch Personen erreichen, die nicht an einer Präsenzveranstaltung hätten teilnehmen können (P.14). Insofern birgt digitale Lehre auch ein inklusives Element.

Die erfolgte Interpretation der Befunde darf die Limitationen der Studie nicht außer Acht lassen. An erster Stelle ist die Selbstselektion bei der Teilnahme an der Webparade zu nennen. Es ist anzunehmen, dass überwiegend solche Lehrenden das Format der Webparade nutzten, die eine digitale Affinität aufweisen und den digitalen Wandel erfolgreich angegangen sind. Lehrende, die Vorbehalte gegenüber digitalen Formaten haben, sich existenziellen finanziellen Schwierigkeiten gegenübersahen oder ihre Trainertätigkeit aufgeben mussten, wurden durch diese Erhebung wohl nicht erreicht.

Eine weitere Limitation des Beitrags stellt der einmalige Erhebungszeitpunkt im Spätsommer des Jahres 2020 dar. Angesichts der sich ständig verändernden politischen, rechtlichen und wirtschaftlichen Rahmenbedingungen für Weiterbildung stellen die Befunde nur eine Momentaufnahme dar, welche durch weitere Forschung ergänzt werden muss, um ein Gesamtbild von Erwachsenen- und Weiterbildung unter Pandemiebedingungen zu ergeben. So sollten die Anforderungen an die Lehrenden sowie an das planende und leitende Weiterbildungspersonal über den weiteren Verlauf und das Ende der Corona-Pandemie hinaus untersucht werden.

Hinsichtlich der Kompetenzentwicklung von Lehrenden lässt sich unter Berücksichtigung der Limitationen dieser Studie ein vorsichtiges positives Fazit ziehen. Die an der Webparade teilnehmenden Lehrenden erkannten eigene Qualifizierungsbedarfe, vor allem im Bereich der digitalen mediendidaktischen Kompetenz, und berichteten, sich entsprechend fortzubilden. Diesbezüglich könnte der geringe Formalisierungsgrad in der Qualifizierung der Lehrenden sogar von Vorteil sein, da sich kommerzielle Train-the-Trainer Angebote vermutlich recht schnell auf die Vermittlung digitaler Kompetenzen umstellten. Zudem berichteten und empfahlen die Lehrenden zahlreiche Bewältigungsstrategien im Umgang mit schwierigen beruflichen Situationen. Da die Digitalisierung nach Angaben der Lehrenden aber nicht nur die didaktische Umstellung, sondern auch die Umstrukturierung ihrer Geschäftsmodelle und -prozesse betrifft, bleibt abzuwarten, wie gut sie mit diesen tiefgreifenden Veränderungen zurechtkommen.

Für die betriebliche Praxis erscheint eine zunehmende Verzahnung von Weiterbildung mit Organisationsentwicklungsstrategien wünschenswert, bei der Lehrende immer mehr als Lernbegleitende fungieren. Die räumliche und zeitliche Flexibilisierung von Lehr-Lernangeboten stellt diesbezüglich eine Chance dar, sodass die beschriebenen Entwicklungen die Wirksamkeit betrieblich-beruflicher Weiterbildung positiv beeinflussen könnten.
